# Narratives on Sexuality in Vulvodynia and Endometriosis: A Qualitative Study on Italian Couples

**DOI:** 10.1007/s10508-026-03507-0

**Published:** 2026-08-15

**Authors:** Elena Fabbri, Giulia Rasolo, Maria Rinaldi, Giacomo Chiara

**Affiliations:** 1 School of Interactionist Psychotherapy, Psychologist and Psychotherapist, Padua, Italy; 2 School of Interactionist Psychotherapy, Psychologist and Psychotherapist, Padua, Italy; 3 School of Interactionist Psychotherapy, Psychologist and Psychotherapist, Padua, Italy; 4 Department of Neuroscience, Reproductive Sciences and Odontostomatology, University of Naples Federico II, Via Pansini, 5, 80131 Naples, Italy

**Keywords:** Sexuality, Vulvodynia, Endometriosis, Relational patterns, Dyspareunia, DSM-5

## Abstract

Although qualitative research on dyspareunia has increased in recent years, most studies have focused on women’s individual experiences or on specific difficulties within the couple, leaving underexplored how partners jointly construct the meanings of sexuality when pain becomes part of the relationship. Against this backdrop, this study explored how couples make sense of sexuality and how their interactional processes shape the relational patterns through which they navigate intimacy in the presence of pain. Drawing on episodic interviews with nine Italian heterosexual couples (M age = 34 years) affected by dyspareunia associated with vulvodynia and/or endometriosis, we conducted a reflexive thematic analysis through which four themes were co-constructed: “Between Body and Emotion: The Shifting Landscape of Sexuality”; “He Holds Back, She Consents: Silent Sacrifices”; “Together Alone: When Coping Is Delegated”; and “From Pain to Partnership: Reimagining Sexuality Together.” Across narratives, couples described greater connection and intimacy when they were able to co-construct meanings and activate communicative spaces grounded in acknowledgment, mutual support, and negotiation. These findings underscore the importance of couple-centered clinical interventions that focus on interactive processes and promote reciprocity and a shared redefinition of sexuality. Adopting a relational and dialogical lens, the study highlights sexuality not as an individual function disrupted by pain, but as an evolving co-created process through which partners continually remake intimacy together.

## Introduction

Vulvodynia is a condition characterized by chronic pain that can be diagnosed in the absence of clinically visible vulvar abnormalities and any evident neurological disease (Bergeron et al., 2020). The symptomatology may involve the entire vulva and/or a specific area of it and is predominantly described in terms of pain (such as deep stinging or a “pins and needles” sensation), but may also include burning sensations, itching, and vulvar discomfort. These symptoms have been associated with disruptions in daily activities and lower levels of psychophysical and sexual well-being (Barnabei, 2020; Queiroz et al., 2024; Santangelo et al., 2023). Despite challenges and delays in diagnosis, the estimated prevalence among women ranges between 7 and 15% (Barnabei, 2020; Bergeron et al., 2020).

Endometriosis is a complex gynecological condition characterized by the growth of endometrium-like tissue outside the uterus (Bulun et al., 2019; Mick et al., 2024). The symptomatology is primarily marked by severe pelvic pain during the menstrual and premenstrual phases (dysmenorrhea) and around ovulation and is often accompanied by chronic physical fatigue and an increased risk of infertility (Fleming & Hardy, 2025; Horne & Missmer, 2022; Pais & Almeida‐Santos, 2023). Pain may also occur during or following sexual intercourse (dyspareunia) (Merli et al., 2024; Wahl et al., 2021; Yong, 2017).

Due to the high variability in symptom presentation, endometriosis is often underdiagnosed or diagnosed only after a significant delay (Bandiera et al., 2009; Davenport et al., 2022; De Corte et al., 2025; Perkins, 2019). According to data from the World Health Organization, it affects approximately 10% of women of reproductive age worldwide (World Health Organization [WHO], 2023). Both endometriosis and vulvodynia are consistently linked to reduced quality of life and difficulties in sexual functioning, intimate relationships, and daily life (Del Forno et al., 2024; Queiroz et al., 2024; Santangelo et al., 2023).

Among the various symptoms is dyspareunia, defined as persistent or recurrent pain experienced during penetration, either at the vulvar level or deeper, which has been linked to disruptions in sexual activity and lower reported relationship quality (Alizadeh & Farnam, 2021; Del Forno et al., 2024; Hill & Taylor, 2021; Merli et al., 2024; Tayyeb & Gupta, 2023). Specifically, levels of sexual arousal, sexual satisfaction, and frequency of intercourse have been found to be significantly lower in women with endometriosis (Di Donato et al., 2014; Fritzer et al., 2013, 2016) or vulvodynia (Desrochers et al., 2008; Masheb et al., 2004; Smith et al., 2011) who report pain during penetrative sex compared to those who do not experience such pain.

However, some studies have found that most women continue to engage in sexual intercourse despite experiencing pain, with the intention of pleasing and fulfilling their partner’s needs, in order to maintain intimacy, and avoid feelings of guilt, shame, inadequacy, or the fear of disappointing or losing their partner (Ayling & Ussher, 2008; Muise et al., 2017; Rosen et al., 2015). The literature shows that many women with dyspareunia tend to suppress pain signals and persist in painful sexual activity (Brauer et al., 2014; Elmerstig et al., 2013) in order to avoid emotional consequences, such as relational or identity-related conflicts (Dewitte et al., 2011; Elmerstig et al., 2008; Engman et al., 2018).

Ekholm et al. (2024) reported that the use of dysfunctional coping strategies—such as avoiding sexual activity or consenting to endure pain—was associated with poorer psychosexual functioning, higher relational catastrophizing, and more negative partner responses. In general, listening to and accommodating each other’s sexual needs and desires are commonly described as strategies associated with higher relationship quality. At the same time, being responsive to a partner’s sexual needs is associated with greater sexual and relationship satisfaction (Vowels et al., 2022).

A substantial body of quantitative dyadic research has examined partner responses and sexual communication in couples coping with dyspareunia. These studies have shown that higher levels of dyadic sexual communication are associated with greater sexual satisfaction and relationship quality for both partners (Pazmany et al., 2014, 2015; Rancourt et al., 2016, 2017). Research on partner responses has further indicated that facilitative responses—those perceived as validating and supportive—are associated with lower pain intensity and better sexual functioning, whereas negative or solicitous responses are linked to greater pain and poorer sexual and relational outcomes (Desrosiers et al., 2008a; Rosen et al., 2012, 2013, 2014, 2015). Daily diary and dyadic studies similarly suggest that collaborative sexual communication patterns are associated with improved sexual adjustment over time (Rancourt et al., 2017; Rosen et al., 2014).

Together, this line of research underscores the central role of interpersonal processes in shaping sexual satisfaction, pain experience, and relational well-being in the context of chronic genital pain. However, these processes are primarily examined through predefined constructs (e.g., communication quality, partner responses, sexual goals) assessed via standardized measures, offering limited insight into how partners themselves make sense of sexual difficulties and position themselves and each other within the relational management of pain and intimacy.

Complementing this quantitative body of work, qualitative studies have begun to explore how couples experience sexual difficulties associated with vulvodynia. For example, Myrtveit‐Stensrud et al. (2023), in a qualitative study based on individual interviews with both partners, highlighted how couples experienced difficulties in communicating about vulvodynia and navigating its consequences within their relational and sexual lives, including avoidance, shame, helplessness, and loneliness.

Overall, the experience of pain is often described as challenging women’s sense of closeness and ability to express affection, often eliciting feelings of guilt, shame, and inadequacy as a sexual partner (Bergeron et al., 2015; Elmerstig et al., 2008; Sheppard et al., 2008). Partners also report similar emotions, including feelings of helplessness, frustration, concern, and anger (Culley et al., 2017), and describe how their partner’s pain experience has a negative impact on the relationship and on their own emotional well-being (Margatho et al., 2022; Smith & Pukall, 2014; Stragapede et al., 2024).

Women with endometriosis who perceive their partner as informed and engaged in their health condition report greater relationship satisfaction (Facchin et al., 2021). This finding suggests that sharing one’s experience and pain condition with the partner may support the joint exploration of coping strategies, fostering a sense of shared responsibility in making decisions and adapting to possible changes (Merli et al., 2024). There also appears to be a high degree of interdependence and mutual influence between partners—both positive and negative—with regard to psychological distress and sexual satisfaction (Schick et al., 2022).

In general, most studies agree on recognizing the impact of these clinical conditions on the sexual lives of partners (Ameratunga et al., 2017; Facchin et al., 2020). Despite sexuality being a key aspect of health, the quality of the relationship with one’s partner has often been insufficiently investigated (Pluchino et al., 2016). Indeed, most of the literature has primarily focused on the experience of the individual in pain, placing less emphasis on the dynamics of the couple relationship (La Rosa et al., 2020).

Only a limited number of qualitative studies have explicitly adopted a couple-based perspective in the context of vulvodynia and endometriosis. For instance, Law et al. (2025), drawing on in-depth interviews with 22 heterosexual couples, examined the multifaceted impact of endometriosis on sex, intimacy, and relational dynamics, highlighting gendered forms of emotion work and strategies for managing sexual difficulties. Similarly, Myrtveit-Stensrud et al. (2025), based on individual interviews with eight heterosexual couples, explored how couples navigate vulvodynia within a fear-avoidance–endurance framework, emphasizing communication challenges, sexual expectations, and patterns of avoidance and endurance. Other qualitative research has focused either on women’s help-seeking experiences and perceptions of partner support (Lountzi & Durand, 2024) or on male partners’ negotiations of masculinity and sexual scripts in the context of vulvodynia (Myrtveit-Stensrud et al., 2025).

While existing qualitative studies have examined dynamics in couples facing dyspareunia associated with vulvodynia and endometriosis, they have primarily focused on emotional distress, coping strategies, communication challenges, or gendered expectations. Even when both partners have been included, analyses have often foregrounded individual experiences or explanatory frameworks (e.g., fear-avoidance processes) rather than systematically examining how partners position themselves and each other across their accounts. As a result, the couple is often treated as a relational context rather than as the primary unit of interpretive analysis.

Consequently, less attention has been devoted to examining how partners jointly construct the meanings of sexuality and navigate intimacy as an ongoing relational process.

The present study shifts the analytic focus from experiences and explanatory models to the relational configurations through which sexuality is organized within the couple, as reconstructed across partners’ parallel narratives. By relational configurations, we refer to recurring patterns of mutual positioning and meaning organization that emerge across partners’ accounts, rather than to isolated behaviors or individual coping strategies. Methodologically, the study adopts a relational analytic approach by examining interviews conducted separately with both partners and analyzing them comparatively, thereby treating the couple as the primary analytic unit and identifying relational patterns through which sexuality is co-constructed within the couple in the presence of pain.

This contribution adopts a theoretical framework that integrates the social constructionist perspective (Gergen, 2023; Romaioli & McNamee, 2021) with the interactionist approach (Mead, 1934; Salvini, 2004; Salvini & Dondoni, 2011), both of which place the relationship and the co-constructed meanings at the center of a semiotic interaction (see Chiara et al., 2023; Chiara & Romaioli, 2021; Romaioli et al., 2023; Salvini, 2011; Sametband et al., 2024). It also draws on the concept of relational patterns—that is, the communicative modes shared within a relational system—and is inspired by the model of patterns in interpersonal interaction (Tomm et al., 2014). In this perspective, the couple is conceptualized as a relational unit of meaning making, rather than as the sum of two individual experiences.

According to these premises, we therefore understand sexuality and relational patterns not as objective phenomena, but as dialogical constructions that emerge within couples’ interactions and in the research encounter itself. The research team, composed of psychologists and psychotherapists trained in the interactionist approach, shares a clinical and theoretical sensitivity toward the dynamics of intimacy, gender, and pain. Our professional background shaped both the questions we asked and the interpretive lenses we applied to participants’ accounts. A reflexive stance was maintained throughout the research process to remain aware of how our theoretical orientation and experiential knowledge contributed to the construction of meanings during analysis.

The research question guiding this study is as follows: How do couples living with vulvodynia and/or endometriosis make sense of sexuality, and how do their interactional processes shape the relational patterns through which they navigate intimacy in the presence of pain?

## Method

### Participants and Procedure

The research was conducted using an episodic interview format (Flick, 2023), administered online due to the COVID-19 restrictions in place in Italy at the beginning of 2022. Episodic interviewing combines conceptual questions with prompts that elicit concrete, situated episodes; this format enables access both to participants’ articulated meanings and to the lived experiences through which those meanings take form.

In designing the interview flow, we were guided by Flick’s recommendations for episodic interviewing, alternating semantic questions with episode-based prompts and encouraging concrete, context-rich descriptions of lived experience.

This approach is well aligned with a relational constructivist perspective, which attends to how understandings emerge within interaction rather than being treated as fixed individual attributes.

The interviews were conceived as dialogical spaces that encouraged participants to articulate and reflect on their personal and relational meanings. The interviewers’ role was to facilitate reflection through open, circular, and exploratory questioning, inviting participants to consider how sexuality and pain were experienced, interpreted, and negotiated within the couple. The interviewers maintained an empathic and reflexive stance, attentive both to the emotional nuances in participants’ accounts and to their own reactions in the unfolding dialogue. This relational posture supported the emergence of narratives that were not predefined but developed through the interview interaction itself.

To operationalize this approach, semantic questions (e.g., “What does sexuality mean to you?”) explored participants’ conceptualizations of sexuality and intimacy; circular questions (Tomm, 1988, 1989; e.g., “What do you think your partner believes about sexual pain?”) invited reflection on intersubjective perspectives; and episodic prompts (e.g., “Tell me about an episode in your sexual relationship where you felt understood and accepted, and one in which you did not.”) elicited concrete situations through which relational patterns and interactional processes could be examined. The full interview guide is provided in Appendix A.

Eighteen interviews were conducted with nine Italian couples by the first three authors under the supervision of the fourth author. All interviews were conducted by women researchers. This is reported as part of the procedural transparency of the study.

The study involved nine couples residing in various Italian regions (Emilia–Romagna, Lombardy, Apulia, Marche, Piedmont, Campania, and Trentino).

Participants were recruited through an online call for participation posted in Italian social media support groups (e.g., Facebook) and on the websites of national associations dedicated to vulvodynia and endometriosis. The call invited couples in which one partner experienced pain during intercourse to take part in the study. Although the announcement did not specify gender or sexual orientation, all respondents who met the criteria self-identified as heterosexual couples.

Individuals who expressed interest contacted the research team via email. Those who confirmed that both partners were willing to participate received detailed information about the study and separate informed consent forms. Individual interviews were scheduled only after written consent was obtained from each partner.

Eligibility criteria required: (1) being in a stable relationship for at least six months; (2) having received a medical diagnosis of vulvodynia and/or endometriosis associated with dyspareunia, as reported by the participant; and (3) willingness of both partners to participate. All participants were adults (18+). Interviews were conducted separately with each partner to promote openness and reduce cross-influence during narration.

Additional background information was collected from participants, including demographic and clinical variables (e.g., onset of symptoms, use of medication, ongoing psychological treatment). These details were collected to support a richer understanding of participants’ contexts and experiences, rather than for analytic comparison. The characteristics of all participants are presented in Table 1, those of women partners in Table 2, and those of men partners in Table 3.

**Table 1:** Demographic characteristics of participants (N = 18)

Characteristic	Value
Mean age of participants	34 years
Mean duration of relationship	6–7 years
Cohabiting couples	4 out of 9
Married couples	2 out of 9
Region of origin	Emilia–Romagna (2); Lombardy (5); Piedmont (3); Apulia (2); Marche (2); Campania (2); Trentino–Alto Adige (2)

**Table 2:** Characteristics of women participants (N = *9)*

Characteristic	Value
Diagnosed condition	Vulvodynia (3); endometriosis (3); both conditions (3)
Mean time since symptom onset	~ 9 years (prior to interview)
Diagnosis timing	Mostly delayed after symptom onset
Pain frequency	Recurrent, especially during intercourse
Pain localization	Mostly during penetrative intercourse
Perceived factors influencing pain	Majority reported additional factors
Pain with previous partners	6 out of 9 reported no pain with previous partners
Psychological/psychotherapeutic treatment	Nearly all currently or previously treated
Medication use	Most take medications related to the condition

**Table 3:** Characteristics of men participants (N = *9)*

Characteristic	Value
Previous partner(s) with painful intercourse	Majority reported none
Presence of sexual dysfunction/disorder	2 out of 9 reported some form of dysfunction
Psychological/psychotherapeutic treatment	Majority had not received treatment
Medication use	Majority did not use medication

Consistent with our reflexive thematic analysis approach, the study did not aim for data saturation but for sufficient information power to address the research aims. The inclusion of nine heterosexual couples (18 interviews) was guided by the criteria proposed by Malterud et al. (2016): The specificity of the sample, the richness and quality of the material, the narrow focus of the study, and the in-depth, case-by-case analysis all supported the adequacy of the sample size.

Data collection and analytic engagement with the material unfolded in a recursive manner over the course of the research process, allowing the team to remain attentive to recurring patterns and variations across participants’ accounts. Through repeated engagement with the dataset, we examined whether additional interviews introduced new relational patterns, extended the interpretive range of existing patterns, or challenged the emerging analytic framework.

Across this process, relational patterns were consistently represented across participants’ narratives, and additional interviews did not substantially extend or reconfigure the interpretive scope of the analysis.

Recent qualitative studies adopting partner-inclusive or couple-based designs in the context of dyspareunia have used similarly sized samples (e.g., Myrtveit-Stensrud et al., 2023, 2025), further supporting the appropriateness of our sampling strategy. Aligned with a constructivist epistemology and the aims of reflexive thematic analysis, our goal was not numerical representativeness but an in-depth exploration of the relational patterns through which couples make sense of sexuality in the presence of pain.

### Data Analysis

The textual material analyzed consisted of transcripts from 18 online, video-recorded interviews, each lasting approximately one hour. Data analysis followed Braun and Clarke’s (2006, 2019, 2022) reflexive thematic analysis and was grounded in the cognitive interactionist and relational constructivist framework guiding the study. Accordingly, the analytic process was conceived as an interpretive and reflexive engagement with the data rather than as a mechanical categorization. Participants’ accounts were approached as situated narratives, and attention was given to how meanings about sexuality, pain, and relational dynamics were articulated within each couple’s discourse.

All transcripts were read multiple times by the first three authors to build shared familiarity with the narratives; the first author also revisited the video recordings to attune to tone, affective nuance, and interactional context. Early analytic notes and memos were produced individually and later brought into shared discussion. This early analytic engagement occurred alongside data collection and informed ongoing reflections on whether additional interviews contributed new insights relevant to the research aims.

Initial coding was then conducted collaboratively by the first and second authors. Working manually, they annotated the transcripts together, using color-coding to cluster segments and trace emerging relational meanings. Partners’ interviews were first examined separately to preserve each perspective and then compared within and across couples to explore relational patterns.

Once a shared coding foundation had been established, the first and second authors independently developed preliminary subthemes and tentative thematic structures. This deliberate separation aimed to preserve interpretive plurality and avoid premature convergence, in line with reflexive thematic analysis.

The subsequent phase involved bringing these independently developed thematic maps into dialogue. These discussions—often lengthy and divergent—played a central role in refining interpretive awareness and deepening the analytic process. Divergences were treated not as discrepancies to be resolved but as analytic resources that expanded the interpretive horizon. Rather than aiming for consensus or objectivity, the team sought to maintain transparency and evolving coherence as interpretations took shape. Themes were iteratively refined through repeated movement between the dataset, coded extracts, and interpretive conversations, attending to coherence across cases and to how researchers’ assumptions shaped meaning making. This recursive process led to the final set of themes presented in the findings (Table 4).

**Table 4:** General themes and subthemes from reflexive thematic analysis

General Themes	Subthemes
Between body and emotion: The shifting landscape of sexuality	Feelings; body; before–after
He holds back, she consents: Silent Sacrifices	Rejection; insecurity; guilt; avoidance; hiding pain
Together alone: When coping is delegated	Individual choices; “it’s your/my problem”
From pain to partnership: Reimagining sexuality together	Relational management; communication; understanding needs

### Researcher Reflexivity and Positionality

The research team included three women interviewers and one senior supervisor, all trained in relational constructivist and interactionist approaches. The gender of the interviewers, alongside their clinical background, may have influenced the interactional dynamics of the interviews and the interpretive process, an aspect that was acknowledged and discussed as part of ongoing reflexive practice.

Reflexivity was an integral part of the analytic process. The first and second authors, who then conducted the analysis, regularly examined how their personal experiences, assumptions, and emotional responses—particularly regarding gender roles, intimacy, and care—could influence interpretive decisions. Divergent viewpoints within the team were treated as analytical resources rather than discrepancies to be resolved, helping to broaden interpretive possibilities and maintain awareness of the partial and situated nature of the findings.

We acknowledge that the configuration of the research team, as well as our shared theoretical orientation, informed both the production and interpretation of the data. These influences are understood not as methodological limitations but as inherent features of qualitative inquiry, shaping the dialogical space through which meanings were co-constructed.

## Results

The reflexive thematic analysis guided the development of four main themes that respond to the research questions and highlight the meanings attributed to sexuality, the prevalent relational patterns, and the strategies enacted by the couples. The themes were constructed through a comparison of the partners’ experiences, in order to emphasize the relational dimension of sexuality within couples facing pain-related conditions.

While themes were not mutually exclusive and individual couples could contribute to multiple patterns, the first three themes were reflected in most couples’ accounts, whereas the final theme was represented in fewer narratives.

### Between Body and Emotion: The Shifting Landscape of Sexuality

The narratives revealed representations that were largely shared between men and women participants, in which sexuality was understood as an experience involving both the body and the affective dimension.

Many of the women described sexuality as something that begins with an emotional bond, which then finds expression in physical contact. Similarly, for several men, the sexual experience was an expression of love and emotional communion, but also of “carnal union” with their partner:

It is something that is very important for the couple. Surely it is the highest expression of the feeling you can have for the other person […] it shouldn’t just be something carnal, but also a feeling. [man, relationship length: 6 years]

The meaning of sexuality appeared strongly connected to the idea of “fusion between bodies,” yet this possibility was disrupted or profoundly altered by the experience of pain. In many women’s accounts, the onset of pain marked a “before” and “after,” a true biographical rupture.

Several women described the contrast between sexuality before the illness and their current experience in terms of loss:

I don’t see anything sexy. I feel like I’m an empty doll. But if I look at pictures from 2016 […] I see life, I see eroticism. Now I don’t see it, I see a façade […] I feel like a broken doll, and this deeply impacts my sexuality. [woman, relationship length: 3 years]

Sexuality, once described as something to be “enjoyed,” was now experienced as “impossible,” at times entirely absent due to pain:

We weren’t able to have intercourse anymore, because it was totally impossible, even just the idea of being penetrated. [woman, relationship length: 4 years]

In some accounts, this change was addressed through a shared redefinition of the meaning of sexuality. Couples who managed to renegotiate sexuality often moved away from the centrality of penetrative intercourse and embraced new ways of experiencing intimacy, such as erotic play, mutual masturbation, affectionate touch, or the use of sex toys. In other accounts, however, the redefinition was asymmetrical: One partner adapted their experience to the new condition, while the other remained anchored to a vision of sexuality tied to penetration or to modes of intimacy that were no longer meaningful within the couple’s current experience. This lack of mutual renegotiation was associated with experiences of misunderstanding and distance, as the meaning of the sexual act was no longer shared or adapted within the couple.

In sum, the meaning of sexuality in the couples interviewed could be profoundly transformed with the onset of pain. For some, this entailed a painful sense of loss; for others, it opened up a space for redefinition and adaptation, in which body and heart, albeit with difficulty, sought new forms of dialogue.

## He Holds Back, She Consents: Silent Sacrifices

This theme captures mirrored, tacit strategies whereby partners restrain themselves or acquiesce in order to protect the relationship, while communication remains constrained.

In couples living with a pain condition, sexuality can become a sensitive space, where gestures and renunciations turn into strategies aimed at preserving the relationship. This pattern is built on silences and implicit readings, in which both partners enact adaptive strategies that, at least initially, aim to avoid hurting the other, but intertwine with a difficulty in openly communicating.

Men commonly reported feelings of insecurity and frustration linked to the difficulty of understanding their partner’s bodily and emotional boundaries. The onset of pain was experienced as a sudden and not always comprehensible change, one that called into question their role within the relationship and sexuality.

One participant described this sense of rejection as follows:

It was a bit difficult for me to approach, because I took it somewhat personally —in the sense that I couldn’t get past the feeling of being rejected. So I thought the problem was me, even though I knew it wasn’t. Over time, I managed to accept it, by talking, by discussing it. Gradually I came to understand that it didn’t… it didn’t depend on her. [man, relationship length: 14 years]

This insecurity led many men to scale back their behavior, holding back or avoiding sexual initiative for fear of seeming inappropriate or invasive:

By now I’ve toned myself down. It’s like I used to be a hot-air balloon, and now I’m a deflated balloon. Given my character, my ability to downsize helps me… let’s say, to support everything, because I have a great spirit of adaptation. But the pain, the frustration, the sadness, the moments of depression — they are there. It feels like being in an increasingly smaller room. [man, relationship length: 3 years]

This avoidance translated into holding back desires, not only physically but also verbally:

I tell her that I would need a bit more closeness, a bit more affection… I’d like to feel her more. But sometimes it’s kind of a “taboo,” in quotes, because maybe she thinks I’m insisting too much on this… but that’s not the case. And so sometimes we avoid it… or rather I avoid talking about it, because I know that on her side there might be a stronger reaction. [man, relationship length: 9 years]

Many women reported feeling pressure to meet their partner’s expectations—whether real or perceived. This pressure took two forms: avoiding physical contact or consenting, even at the expense of their own desire. Most women reported hiding or minimizing their pain during intercourse in order to safeguard the intimate dimension of the couple:

When we do manage to have intercourse, I try not to let him notice too much, you know? Sometimes, even if I’m in pain, I try to hide it from him. If one position causes me more pain, I try… unless it becomes unbearable, then no. But when I can manage it, I sometimes try not to let him realize that at that moment, that particular position hurts me. But it does have an impact. It affects us a lot because… it limits our desire, our passion for each other. [woman, relationship length: 2 years]

A sense of guilt was recurrent in women’s narratives, often linked to feeling inadequate when they perceived they could not meet their partner’s desires. Statements such as “I feel guilty for having to hold him back” reveal an internal conflict between listening to one’s own body and the fear of disappointing the partner or losing the relationship. In some accounts, this feeling even fueled the fear of possible infidelity.

This theme portrays a restrained and silent intimacy. Often both partners adopted tacit and protective behaviors (“I don’t say I’m in pain,” “I don’t say I have desire”) in order not to hurt the other. They moved with caution, frequently in a mirrored way: He held back so as not to seem insistent, while she consented or concealed in order not to disappoint him.

The pattern that developed was one of parallel restraint, oriented toward the other, with each partner seeking to protect the other in silence. Desire was not confronted, but interpreted. It was not asked for, but renounced. Pain was not shared, but hidden.

This movement seemed to allow the couple to maintain closeness, through the sense of having found a solution that, however, did not appear to be sustainable for either partner or for the couple. It represented an attempted management of difficulties without clear communication, where the strategies adopted—though driven by the intention of not hurting the other—constrained the possibility of authentic and shared intimacy.

## Together Alone: When Coping Is Delegated

This theme captures unilateral, explicitly verbalized attempts by one partner to “solve” sexual difficulties alone. Unlike the mirrored and tacit coordination described above, strategies here were enacted individually and articulated openly. Participants portrayed these moves as well-intentioned yet often increasing distance when not co-constructed. The relational pattern that developed was one of unilateral coping: The problem was perceived as individual, and the search for a response likewise remained individual. Although motivated by positive intentions, these strategies often became attempts at solutions that were not shared, ultimately generating frustration for both partners.

One man, for example, recounted having asked his partner to engage in role-play with the intention of making her feel desired. However, this proposal stemmed from his own interpretation that she “did not feel like a woman,” rather than from an open dialogue with her. The attempt, though well intentioned, became laden with expectations and turned into an unwanted form of pressure:

I tried, maybe in the simplest way that came to me — role-playing. Even though I had no idea how to do it, so I probably did it in a bit of a naïve way. But always with the aim of making her feel much more like a woman. […] I tried in every way, but it was useless. To… to get her to spark, I don’t know how else to put it. [man, relationship length: 3 years]

The role-play proposal, intended by him to make her feel more desired, instead placed her under pressure, accentuating her sadness and withdrawal, while he, faced with rejection, felt powerless and devalued in his affective role. Such a solitary strategy, rather than generating intimacy, was described as accentuating emotional distance and leaving the partner with the feeling of “not being enough.”

Similarly, some women described acting for their partner but without the partner, thereby keeping solutions individual rather than shared.

One woman reported buying a sex toy with the explicit request that he use it alone:

I bought it for him, but he has to use it by himself, that’s it. I don’t want to know anything about it, I don’t want to see it… It’s something for him, not for us. [woman, relationship length: 3 years]

Although conceived as a caring gesture that acknowledged a need, this choice became a separating strategy, not only avoiding shared intimacy but also risking reinforcing that separation. In some situations, instead of seeking alternatives or initiating dialogue, one partner passively delegated responsibility to the other. A man, referring to his partner, stated:

I really don’t know how to move forward, so you have to give me the starting point because right now I’m really at a loss. [man, relationship length: 7 years]

This stance appeared to shift the emotional and decision-making burden onto the partner already living with the pain condition, generating feelings of loneliness and pressure. As a woman described:

Inevitably, it’s all tied to endometriosis. So inevitably, the problem is me. As if I also had to find the right way forward […] But I ask him repeatedly, like, help me understand what could be an alternative… but with no result. […] And this is frustrating for me because it feels like I have to find the solution on my own — one that should work for both of us. [woman, relationship length: 14 years]

Delegating unilateral coping was associated with frustration and emotional distance, compromising the relational dimension of sexuality by treating it as a solitary task rather than a space for mutual sharing.

## From Pain to Partnership: Reimagining Sexuality Together

In contrast, in accounts where the problem was seen as a couple’s issue rather than as one belonging solely to the partner with the condition, a pattern of mutuality and negotiation emerged, where suffering was recognized as shared and sexuality as a domain to be reimagined together. One man expressed it in these terms:

In my opinion, we need to join forces… and for that, I think the man’s side also needs to play a role… because both of us are suffering… it’s not just one person who suffers. [man, relationship length: 4 years]

In some accounts, the onset of pain in the sexual sphere did not represent an insurmountable obstacle, but rather the beginning of a shared journey of renegotiation. When sexuality as traditionally understood—centered on penetrative intercourse—became painful or impracticable, partners described exploring new ways of living intimacy. In these narratives, change was not passively endured but approached with creativity, dialogue, and mutual support.

In these accounts, constructing a new intimacy together involved open communication and the legitimization of each partner’s needs. Desire, pain, and frustration were named and shared rather than avoided. In this process, partners learned to recognize their own limits and those of the other, without assigning blame or holding implicit expectations. As one participant explained, the very possibility of talking openly about rejection or distance allowed for the recovery of a deeper emotional connection:

For example, sometimes we’ve talked about the rejection he feels when I don’t seek him sexually… and actually, this created greater closeness. Basically, when there’s withdrawal, it’s as if we become emotionally distant… but when there’s a strong emotional connection, there’s also a kind of release… of our mental inhibitions. So in that moment, we might even be more willing to talk about our fantasies… to use external means to increase my sexual desire or his. [woman, relationship length: 6 years]

In this context, sexuality was progressively decentered from penetration and reformulated through the exploration of alternatives: erotic play, mutual masturbation, caresses, the use of sex toys, or even simply moments of affectionate physical contact. These strategies were not seen as second-best solutions but as conscious choices that enriched the relationship and fostered a deeper understanding of one another:

We changed many things — our approach, we started using sex toys, playing more with our bodies — and it was also positive because we got to know each other better. So in a certain sense, things changed positively, leaving penetration aside. [woman, relationship length: 10 years]

This mutual stance was also evident in everyday gestures: in the ability to wait, not to pressure, and to communicate desire without imposing it. Some women described attentive and gentle partners who, while expressing desire, conveyed closeness without exerting pressure:

He becomes maybe more delicate, more attentive, and maybe stays there, waiting for me to take the initiative more than usual […]. But he always makes me feel desired, that he desires me — he never lets me miss that. [woman, relationship length: 2 years]

Beyond the erotic dimension, intimacy was also built through the feeling of being seen, accepted, and acknowledged in one’s needs. It was not necessary for desire always to be fulfilled: Sometimes simply having it legitimized and considered was enough to create a sense of closeness. As one participant affirmed:

I think I’m lucky to have a good relationship with him, both psychologically and emotionally. I can somehow prioritize his desires as well. […] I think being able to put yourself in your partner’s shoes can only be a positive thing. [woman, relationship length: 9 years]

In this context, prioritizing the partner’s desires referred to recognizing his needs for intimacy and sexual expression within the relationship, while continuing to negotiate forms of closeness that remained compatible with the experience of pain.

These narratives show that the renegotiation of sexuality was more than a simple adaptation to a painful condition—it was an opportunity for relational growth. In accounts where new forms of contact and communication were constructed together, intimacy was transformed into a co-constructed space, flexible and resilient, where desire could find new ways of being expressed. Within this dynamic, a relational pattern of negotiated mutuality emerged: Sexuality was no longer taken for granted but was built through dialogue, recognition of each other’s needs, and the sharing of difficulties.

## Discussion

This qualitative study contributes to the growing body of research (e.g., Facchin et al., 2021; Law et al., 2025; Myrtveit-Stensrud et al., 2025) examining sexuality in the context of dyspareunia associated with conditions such as vulvodynia and endometriosis, by adopting an explicitly relational and meaning-centered lens.

Rather than focusing solely on the individual woman’s pain experience, the study situates sexuality within the couple’s ongoing negotiation of intimacy and shared meanings (Gergen, 2023; Tomm, 1988).

In doing so, it complements and problematizes biomedical and individualistic framings that locate pain and sexuality within the woman’s body, highlighting instead that sexual experience emerges from relational processes of negotiation and shared meaning making. This relational reorientation deepens theoretical understanding and offers a framework for rethinking how sexuality, when disrupted by pain, can be addressed as a shared and evolving process rather than as an individual dysfunction.

Through the reflexive thematic analysis, four themes were co-constructed, each illuminating a different configuration of how couples make sense of sexuality in the presence of pain.

The first theme, “Between Body and Emotion: The Shifting Landscape of Sexuality,” highlights how sexuality was narrated as an intertwining of bodily and emotional dimensions.

As Romaioli and Caprini (2025) have noted, experiences of illness and pain are embedded in relational contexts that shape how individuals make sense of their condition. This echoes previous findings that pain can reshape meanings of self and sexuality within intimate relationships (Ayling & Ussher, 2008).

Our findings articulate and extend these perspectives within the sexual domain, showing how pain disrupts the sense of bodily continuity that sustains intimacy and reconfigures sexual subjectivity, transforming desire into vigilance and pleasure into fear of hurting or being hurt. In this sense, the “before and after” described by participants can be read as a shift in embodied meaning making, from a spontaneous experience of connection to one that requires ongoing negotiation and reinterpretation. Sexuality thus appears as a dialogical construction, in accounts where meanings were co-constructed—moving beyond the centrality of penetration—sexuality was described as an affective and exploratory domain rather than a lost function. Conversely, where meanings remained rigid or asymmetrical, the possibilities for adaptation appeared more constrained, and intimacy tended to take the form of avoidance or frustration.

This dynamic can be interpreted in light of literature conceptualizing penetrative intercourse as a dominant heterosexual sexual script, which structures expectations about what counts as sex within intimate relationships (Simon & Gagnon, 1986, 2003), as well as more recent work suggesting that greater flexibility in sexual scripts may support couples’ sexual well-being when facing sexual challenges (Bouchard et al., 2023).

The renegotiation of what sexuality means within the couple appeared as a key relational resource for resilience, allowing partners to integrate bodily limitation into a new shared narrative of closeness. This interpretation expands prior conceptualizations of sexual adjustment as merely behavioral, reframing it as a process of relational sense making.

The second theme, “He Holds Back, She Consents: Silent Sacrifices,” describes mirrored, tacit strategies through which partners restrained themselves or acquiesced in silence to protect the relationship.

In several accounts, men described holding back physical initiative or silencing desire for fear of appearing intrusive, while women described consenting to intercourse or minimizing pain to preserve closeness. As in previous studies, such dynamics were underpinned by feelings of guilt, shame, and inadequacy (Bergeron et al., 2015; Elmerstig et al., 2008; Sheppard et al., 2008). Within this apparent mutual protection, the sexual relationship appeared to be maintained through what might be called silent reciprocity: a balance built on unspoken renunciations rather than genuine sharing.

This dynamic mirrors what Ayling and Ussher (2008), Brauer et al. (2014), and Muise et al. (2017) have described as women’s tendency to engage in sexual activity despite pain to avoid disappointing the partner, yet our findings extend this perspective by showing how men, too, participate in this unspoken choreography through restraint and self-censorship.

As Helfenstein (2023) noted, men may underestimate how frequently women consent to sexual activity despite discomfort; our participants’ accounts suggest that such misperceptions are part of a broader relational pattern in which both partners attempt to protect the other’s feelings at the expense of their own authenticity.

Across narratives, intimacy appeared to hinge on a fragile balance between protection and authenticity—a paradox at the heart of relational life when pain becomes part of it. These reciprocal silences illustrate how efforts to maintain relational harmony may paradoxically reinforce distance: Desire is no longer expressed but interpreted, and pain is no longer shared but hidden. Both partners become actors in a protective system that prevents genuine negotiation of intimacy.

Some participants attempted to “solve” sexual difficulties, through proposals such as role-playing or by suggesting that the partner find personal outlets for sexual needs. These initiatives, while well intentioned, marked a shift from relational to individual responsibility.

In several accounts, the delegation assumed a gendered meaning, as women were implicitly held responsible for resolving what was framed as their own bodily problem. In these situations, the responsibility for finding ways to manage the impact of pain on sexual activity appeared to fall primarily on the partner experiencing the condition. Some men described feeling uncertain about how to act and implicitly delegating the search for solutions to their partner. Such positioning may intensify feelings of isolation, as the burden of finding solutions is experienced as primarily individual rather than relational. Such dynamics echo sociological analyses of the “labor of love,” which describe how women may become responsible for managing sexual difficulties and sustaining relational intimacy within heterosexual relationships (Cacchioni, 2007). The third theme, “Together Alone: When Coping Is Delegated,” captures moments when one partner handed over the task of finding solutions to the other. The man who proposed role-play, for instance, sought to “make her feel like a woman” but the initiative was described as becoming a source of pressure and emotional distance. Similarly, when a woman described buying a sex toy for her partner to use alone, her gesture acknowledged his needs but reinforced separateness rather than fostering mutual adaptation.

Such unilateral coping transforms sexuality into a problem to be fixed rather than a relational experience to be reimagined together. Meaning making becomes asymmetrical, and responsibility for managing pain or desire is displaced onto one partner. In these accounts, sexuality loses its dialogical quality and becomes a solitary task, undermining the sense of mutuality that sustains intimacy.

These relational patterns show that the challenge is not simply the presence of pain but how couples make sense of it together. The transition toward renegotiation thus begins not with the disappearance of pain, but with the recovery of a shared space where meanings can once again be co-constructed.

The fourth theme, “From Pain to Partnership: Reimagining Sexuality Together,” captures how, in some accounts, sexual difficulties were described as being transformed into a shared experience rather than an individual problem. As one participant expressed, both partners “need to join forces,” recognizing that “both of us are suffering.” In these narratives, pain was described not only as a limitation but also as a starting point for joint reflection, in which intimacy was gradually redefined as a co-constructed and adaptive process.

Our findings suggest that the possibility of a satisfying sexuality does not stem from the resolution of pain but from engagement, within the couple, in processes of relational renegotiation. By negotiation we do not refer to explicit bargaining or strategic compromise, but to an ongoing process of mutual adjustment and reinterpretation through which partners redefine what sexuality means within their relationship. Within this process, communication served not as a mere exchange of information but as a dialogical space where needs, fears, and boundaries could be mutually recognized and reinterpreted. Desire, avoidance, and suffering were not simply expressed but made sense of together.

This interpretive work was described as enabling the reframing of sexuality as an evolving project rather than a lost function.

These results extend previous research that has mainly emphasized individual coping strategies in women (Ekholm et al., 2024; Elmerstig et al., 2013) or the impact of pain on partners (Margatho et al., 2022; Smith & Pukall, 2014). More recent qualitative work has begun to highlight the interpersonal dimensions of genito-pelvic pain, including couples’ efforts to maintain intimacy through communication, adaptation, and the exploration of alternatives to penetrative intercourse (Brown, 2023), as well as women’s coping strategies in relational contexts (Banaei et al., 2023).

However, these studies have primarily described either individual coping or relational adjustments, without systematically examining how meanings, responsibilities, and positions are co-constructed across partners’ narratives.

Our analysis moves beyond this dyadic separation by showing how the couple itself can become a generative context for redefining what intimacy means. It is neither solely the woman who must adapt nor solely the partner who must provide support; rather, it is the mutual activation of a reflective process that opens possibilities for change.

Communication was constructed as pivotal in this process, yet not all forms were equally constructive. In some narratives, talking about sexuality was described as sometimes reinforcing asymmetries when guided by guilt or the search for solutions, whereas in others, dialogue took on a reflexive quality that made space for both partners’ experiences.

This aligns with Alizadeh and Farnam’s (2021) findings that effective communication can mediate the impact of chronic pain on intimacy, but our study suggests a further step: Communication does not merely mediate relationships; it constitutes them.

Similarly, our results resonate with recent work on responsiveness to sexual needs (Vowels et al., 2022), highlighting how the recognition of the partner’s needs—rather than their immediate fulfillment—can sustain connection and mutuality. Even when desire could not always find expression, the act of legitimizing it, of making it speakable, restored a sense of being seen and understood.

In light of prior literature that has primarily focused either on women’s experiences (Ayling & Ussher, 2008; Elmerstig et al., 2013) or on quantitative analyses of the couple (Hämmerli et al., 2018; Rosen et al., 2015), our study also sought to give voice to men’s perspectives, which remain relatively underrepresented. Men’s narratives revealed complex experiences of helplessness, insecurity, and difficulty in understanding their partner’s boundaries, echoing and extending findings by Myrtveit-Stensrud (2023, 2025). By including both partners’ voices, our analysis advances a more symmetrical and relational understanding of how dyspareunia affects sexuality.

While this study offers new insights into how couples negotiate intimacy in the presence of pain, several limitations should be considered when interpreting the findings. Conducting interviews online during the COVID-19 pandemic allowed participants to engage from familiar and protected settings, but it also constrained the possibility of observing the nonverbal or interactive dynamics that might emerge in a shared space. The choice to interview partners separately, although intended to facilitate openness and autonomy of expression, inevitably reduced the opportunity to capture dialogical exchanges in real time, an aspect that would be valuable to explore through couple interviews or observational designs in future research. The fact that all interviews were conducted by women researchers may have influenced how participants articulated their experiences of sexuality. While this influence cannot be determined, it remains a relevant consideration for qualitative interpretation.

Moreover, the sample consisted exclusively of heterosexual couples who had chosen to remain together despite difficulties. This self-selection may have shaped the narratives toward more cohesive or resilient representations of intimacy, leaving unexplored the experiences of couples who separated or of those in non-heterosexual relationships.

Future studies could extend the scope of inquiry by including more diverse relational configurations and by adopting longitudinal or participatory approaches to trace how meanings of sexuality evolve over time. Further research could also examine how relational patterns of meaning making in sexuality may vary across different relationship contexts (e.g., relationship duration or marital status), and how sexuality is embedded within broader domains of couple functioning, such as communication and decision-making processes.

In this sense, couple-based and action research methodologies could be particularly valuable, as they allow both partners to engage simultaneously in processes of change. Interestingly, some participants described the interviews themselves as a reflective space that helped them to talk differently about intimacy, suggesting that even within research contexts, relational reflexivity can become transformative. This observation underscores a key implication of the study: Fostering dialogue about sexuality is itself a form of therapeutic practice.

Clinically, these findings suggest that therapeutic work should not focus solely on symptom management or individual coping, but on how sexuality is organized within the relational dynamics of the couple. Interventions may benefit from exploring how partners position themselves and each other in relation to pain, desire, and the implicit distribution of responsibility for sustaining intimacy. In particular, supporting couples in making explicit unspoken expectations, asymmetries in responsibility, and patterns of mutual restraint may help shift sexuality from an individual problem to be managed to a shared relational process to be co-constructed.

These interpretations are inevitably shaped by our own positioning within a social constructivist framework, which guides both the analytic lens and the attention to the dialogical nature of meaning making. Recognizing this stance allows us to view the findings not as neutral representations but as co-constructed understandings of couples’ lived experiences.

This study contributes to the existing literature by reframing sexuality in couples facing vulvodynia and endometriosis as a relational and meaning making process rather than an individual domain disrupted by pain. While previous research has largely focused on women’s experiences of pain or on biomedical and psychological determinants, our findings foreground the couple as a dialogical context in which sexuality and coping strategies are continuously co-constructed and negotiated. Methodologically, the study introduces a relational lens that approaches the couple as the primary unit of analysis, highlighting the interpretive interplay between partners’ narratives.

In this perspective, sexuality is not a function to be restored but an evolving relational dialogue through which partners continuously reimagine intimacy—transforming pain from a limit into a language of connection.

## Data Availability

Full interview transcripts and coding tables are not publicly available due to ethical constraints and the sensitive nature of the data. However, the article provides a detailed description of the analytic process, including examples of coding and verbatim participant quotations, to ensure transparency. Data and materials are available upon request directly from the corresponding author.

## Appendix A: Full Interview Guide

The guide combined semantic questions exploring conceptual understandings of sexuality and pain, circular questions inviting reflection on the partner’s perceived perspectives, and episodic prompts eliciting concrete narratives of lived experience. The interviewer used the guide to facilitate dialogue, allowing participants to introduce themes, clarify meanings, and recount episodes they deemed relevant. This use of the guide ensured consistency across interviews while remaining responsive to emergent content and relational dynamics.

What does “sexuality” mean to you?

How do you experience sexuality within your relationship?

What do you think your partner believes about sexuality in your relationship?

In what way do you think your mutual beliefs about one another influence your sexual behavior?

When would you consider your sexual life as a couple to be satisfying?

When do you think your partner would consider it to be satisfying?

Tell me about an episode in your sexual relationship where you felt understood and accepted, and one in which you did not.

How has sexual pain influenced or does it influence your sexuality?

What do you think your partner believes about sexual pain?

At this point, is there anything you would change about your sexual life as a couple?

How do you think this change could take place?

What do you think your partner would want to change?

Is there anything that hasn’t come up yet that you feel is important to express your point of view more fully?

## References

[CR1] Alizadeh, A., & Farnam, F. (2021). Coping with dyspareunia, the importance of inter and intrapersonal context on women’s sexual distress: A population-based study. *Reproductive Health,**18*. 10.1186/s12978-021-01206-8 PMC8320204 34321034

[CR2] Ameratunga, D., Flemming, T., Angstetra, D., Ng, S. K., & Sneddon, A. (2017). Exploring the impact of endometriosis on partners. *Journal of Obstetrics and Gynaecology Research,**43*(6), 1048–1053. 10.1111/jog.13325 28621048

[CR3] Ayling, K., & Ussher, J. M. (2008). “If sex hurts, am I still a woman?” The subjective experience of vulvodynia in hetero-sexual women. *Archives of Sexual Behavior,**37*, 294–304. 10.1007/s10508-007-9204-1 17876696

[CR4] Banaei, M., Mehrnoush, V., Roozbeh, N., & Kariman, N. (2023). Coping strategies with genito‐pelvic pain/penetration disorder: A qualitative study. *Pain Research and Management,**2023*(1), Article 5791751. 10.1155/2023/5791751 38144227 PMC10748719

[CR5] Bandiera, S., Cavallaro, A., Aloisi, A., Vitale, S. G., Morello, R., Matarazzo, M. G., Giunta, G., Raciti, G., Rapisarda, F., & Cianci, A. (2009). Management dell’endometriosi: Diagnosi e Terapia. *Giornale Italiano di Ostetricia e Ginecologia,**31*(6/7), 311–317.

[CR6] Barnabei, V. M. (2020). Vulvodynia. *Clinical Obstetrics and Gynecology,**63*(4), 752–769. 10.1097/GRF.0000000000000576 33074981

[CR7] Bergeron, S., Corsini-Munt, S., Aerts, L., Rancourt, K., & Rosen, N. O. (2015). Female sexual pain disorders: A review of the literature on etiology and treatment. *Current Sexual Health Reports,**7*, 159–169. 10.1007/s11930-015-0053-y

[CR8] Bergeron, S., Reed, B. D., Wesselmann, U., & Bohm-Starke, N. (2020). Vulvodynia. *Nature Reviews Disease Primers,**6*(1), 36. 10.1038/s41572-020-0164-2 32355269

[CR9] Bouchard, K. N., Cormier, M., Huberman, J. S., & Rosen, N. O. (2023). Sexual script flexibility and sexual well-being in long-term couples: A dyadic longitudinal study. *Journal of Sexual Medicine,**20*(7), 945–954. 10.1093/jsxmed/qdad067 37280188

[CR10] Brauer, M., Lakeman, M., van Lunsen, R., & Laan, E. (2014). Predictors of task‐persistent and fear‐avoiding behaviors in women with sexual pain disorders. *Journal of Sexual Medicine,**11*(12), 3051–3063. 10.1111/jsm.12697 25234926

[CR11] Braun, V., & Clarke, V. (2006). Using thematic analysis in psychology. *Qualitative Research in Psychology,**3*(2), 77–101. 10.1191/1478088706qp063oa

[CR12] Braun, V., & Clarke, V. (2019). Reflecting on reflexive thematic analysis. *Qualitative Research in Sport, Exercise & Health,**11*(4), 589–597. 10.1080/2159676X.2019.1628806

[CR13] Braun, V., & Clarke, V. (2022). Conceptual and design thinking for thematic analysis. *Qualitative Psychology,**9*(1), 3–26. 10.1037/qup0000196

[CR14] Brown, K. (2023). EndoCouples: Working with couples struggling with painful sex due to endometriosis. *Journal of Sexual Medicine,**20*(Supplement_3), Article qdad068-034. 10.1093/jsxmed/qdad068.034. (035).

[CR15] Bulun, S. E., Yilmaz, B. D., Sison, C., Miyazaki, K., Bernardi, L., Liu, S., Kohlmeier, A., Yin, P., Milad, M., & Wei, J. (2019). Endometriosis. *Endocrine Reviews,**40*(4), 1048–1079. 10.1210/er.2018-00242 30994890 PMC6693056

[CR16] Cacchioni, T. (2007). Heterosexuality and ‘the labour of love’: A contribution to recent debates on female sexual dysfunction. *Sexualities,**10*(3), 299–320. 10.1177/1363460707078320

[CR17] Chiara, G., & Romaioli, D. (2021). The challenge of migratory flows in the Mediterranean Sea to psychology: A single case study from a social constructionist perspective. *Journal of Constructivist Psychology,**34*(1), 98–115. 10.1080/10720537.2019.1701591

[CR18] Chiara, G., Romaioli, D., & Contarello, A. (2023). Self-positions and narratives facilitating or hindering posttraumatic growth: A qualitative analysis with migrant women of Nigerian descent survivors of trafficking. *Psychological Trauma: Theory, Research, Practice, and Policy,**15*(6), 1041–1050. 10.1037/tra0001245 35511544

[CR19] Culley, L., Law, C., Hudson, N., Mitchell, H., Denny, E., & Raine-Fenning, N. (2017). A qualitative study of the impact of endometriosis on male partners. *Human Reproduction,**32*(8), 1667–1673. 10.1093/humrep/dex221 28637285 PMC5850214

[CR20] Davenport, S., Smith, D., & Green, D. J. (2022). Barriers to a timely diagnosis of endometriosis: a qualitative systematic review. *Obstetrics & Gynecology*. 10.1097/AOG.0000000000005255 37441792

[CR21] De Corte, P., Klinghardt, M., von Stockum, S., & Heinemann, K. (2025). Time to diagnose endometriosis: Current status, challenges and regional characteristics—A systematic literature review. *BJOG: An International Journal of Obstetrics & Gynaecology,**132*(2), 118–130. 10.1111/1471-0528.17973 39373298 PMC11625652

[CR22] Del Forno, S., Raspollini, A., Doglioli, M., Andreotti, A., Spagnolo, E., Lenzi, J., Borghese, G., Raimondo, D., Arena, A., Rodriguez, E., Hernandez, A., Govoni, F., & Seracchioli, R. (2024). Painful sexual intercourse, quality of life and sexual function in patients with endometriosis: Not just deep dyspareunia. *Archives of Gynecology and Obstetrics,**310*(4), 2091–2100. 10.1007/s00404-024-07643-7 39052076 PMC11392973

[CR23] Desrochers, G., Bergeron, S., Landry, T., & Jodoin, M. (2008). Do psychosexual factors play a role in the etiology of provoked vestibulodynia? A critical review. *Journal of Sex & Marital Therapy,**34*(3), 198–226. 10.1080/00926230701866083 18398760

[CR24] Desrosiers, M., Bergeron, S., Meana, M., Leclerc, B., Binik, Y. M., & Khalifé, S. (2008). Psychosexual characteristics of vestibulodynia couples: Partner solicitousness and hostility are associated with pain. *Journal of Sexual Medicine,**5*(2), 418–427. 10.1111/j.1743-6109.2007.00705.x 18086164

[CR25] Dewitte, M., Van Lankveld, J., & Crombez, G. (2011). Understanding sexual pain: A cognitive-motivational account. *Pain,**152*(2), 251–253. 10.1016/j.pain.2010.10.051 21122993

[CR26] Di Donato, N., Montanari, G., Benfenati, A., Monti, G., Bertoldo, V., Mauloni, M., & Seracchioli, R. (2014). Do women with endometriosis have to worry about sex? *European Journal of Obstetrics & Gynecology and Reproductive Biology,**179*, 69–74. 10.1016/j.ejogrb.2014.05.022 24965983

[CR27] Ekholm, E., Engman, L., Ter Kuile, M. M., & Flink, I. K. (2024). Coping with provoked vestibulodynia in a relational context—A cluster analysis of coping patterns and their associations with relational cognitions and goals. *European Journal of Pain*. 10.1002/ejp.2297 38822690

[CR28] Elmerstig, E., Wijma, B., & Berterö, C. (2008). Why do young women continue to have sexual intercourse despite pain? *Journal of Adolescent Health,**43*(4), 357–363. 10.1016/j.jadohealth.2008.02.011 18809133

[CR29] Elmerstig, E., Wijma, B., & Swahnberg, K. (2013). Prioritizing the partner’s enjoyment: A population-based study on young Swedish women with experience of pain during vaginal intercourse. *Journal of Psychosomatic Obstetrics & Gynecology,**34*(2), 82–89. 10.3109/0167482X.2013.793665 23701494

[CR30] Engman, L., Flink, I. K., Ekdahl, J., Boersma, K., & Linton, S. J. (2018). Avoiding or enduring painful sex? A prospective study of coping and psychosexual function in vulvovaginal pain. *European Journal of Pain,**22*(8), 1388–1398. 10.1002/ejp.1227 29635880

[CR31] Facchin, F., Buggio, L., & Saita, E. (2020). Partners’ perspective in endometriosis research and treatment: A systematic review of qualitative and quantitative evidence. *Journal of Psychosomatic Research,**137*, Article 110213. 10.1016/j.jpsychores.2020.110213 32781264

[CR32] Facchin, F., Buggio, L., Vercellini, P., Frassineti, A., Beltrami, S., & Saita, E. (2021). Quality of intimate relationships, dyadic coping, and psychological health in women with endometriosis: Results from an online survey. *Journal of Psychosomatic Research,**146*, Article 110502. 10.1016/j.jpsychores.2021.110502 33932718

[CR33] Fleming, A., & Hardy, A. (2025). Endometriosis is more than a painful period. Period. *Journal for Nurse Practitioners,**21*(1), Article 105232. 10.1016/j.nurpra.2024.105232

[CR34] Flick, U. (2023). *An introduction to qualitative research* (7th ed.). Sage.

[CR35] Fritzer, N., Haas, D., Oppelt, P., Hornung, D., Wölfler, M., Ulrich, U., Fischerlehner, G., Sillem, M., & Hudelist, G. (2013). More than just bad sex: Sexual dysfunction and distress in patients with endometriosis. *European Journal of Obstetric & Gynecology and Reproductive Biology,**169*(2), 392–396. 10.1016/j.ejogrb.2013.04.001 23642970

[CR36] Fritzer, N., Tammaa, A., Haas, D., Oppelt, P., Renner, S., Hornung, D., Wölfler, M., Ulrich, U., & Hudelist, G. (2016). When sex is not on fire: A prospective multicentre study evaluating the short-term effects of radical resection of endometriosis on quality of sex life and dyspareunia. *European Journal of Obstetrics & Gynecology and Reproductive Biology,**197*, 36–40. 10.1016/j.ejogrb.2015.11.007 26704015

[CR37] Gergen, K. (2023). *An invitation to social construction*. SAGE Publications Ltd.

[CR38] Hämmerli, S., Schwartz, A. S. K., Geraedts, K., Imesch, P., Rauchfuss, M., Wölfler, M. M., et al. (2018). Does endometriosis affect sexual activity and satisfaction of the man partner? A comparison of partners from women diagnosed with endometriosis and controls. *Journal of Sexual Medicine,**15*(6), 853–865. 10.1016/j.jsxm.2018.03.087 29706579

[CR39] Helfenstein, F., Kohl Schwartz, A. S., Imesch, P., Rauchfuss, M., Wölfler, M. M., Haeberlin, F., von Orelli, S., & Leeners, B. (2023). Comparison of male and female perspective in couples involved in sexual relationships and facing endometriosis. *Sexual Medicine,**11*(2), Article qfad013. 10.1093/sexmed/qfad013 37256216 PMC10226817

[CR40] Hill, D. A., & Taylor, C. A. (2021). Dyspareunia in women. *American Family Physician,**103*(10), 597–604. 33983001

[CR41] Horne, A. W., & Missmer, S. A. (2022). Pathophysiology, diagnosis, and management of endometriosis. *BMJ*, *379*. 10.1136/bmj-2022-070750 36375827

[CR42] La Rosa, V. L., De Franciscis, P., Barra, F., Schiattarella, A., Tropea, A., Tesarik, J., Shah, M., Kahramanoglu, I., Marques Cerentini, T., Ponta, M., & Ferrero, S. (2020). Sexuality in women with endometriosis: A critical narrative review. *Minerva Medica,**111*(1), 79–89. 10.23736/S0026-4806.19.06299-2 31726815

[CR43] Law, C., Hudson, N., Mitchell, H., Culley, L., & Norton, W. (2025). ‘You feel like you’re drifting apart’: A qualitative study of impacts of endometriosis on sex and intimacy amongst heterosexual couples. *Sexual and Relationship Therapy*, *40*, 142–165. 10.1080/14681994.2024.2306316

[CR44] Lountzi, A. Z., & Durand, H. (2024). Help-seeking experiences and intimate partner support in vulvodynia: A qualitative exploration. *Women’s Health,**20*, Article 17455057241241866. 10.1177/17455057241241866 38554074 PMC10981854

[CR45] Malterud, K., Siersma, V. D., & Guassora, A. D. (2016). Sample size in qualitative interview studies: Guided by information power. *Qualitative Health Research,**26*(13), 1753–1760. 10.1177/1049732315617444 26613970

[CR46] Margatho, D., Makuch, M. Y., & Bahamondes, L. (2022). Experiences of male partners of women with endometriosis-associated pelvic pain: A qualitative study. *European Journal of Contraception & Reproductive Health Care,**27*(6), 454–460. 10.1080/13625187.2022.2097658 35867527

[CR47] Masheb, R. M., Lozano-Blanco, C., Kohorn, E. I., Minkin, M. J., & Kerns, R. D. (2004). Assessing sexual function and dyspareunia with the Female Sexual Function Index (FSFI) in women with vulvodynia. *Journal of Sex & Marital Therapy,**30*(5), 315–324. 10.1080/00926230490463264 15672599

[CR48] Mead, G. H. (1934). *Mind, self, and society from the standpoint of a social behaviorist*. University of Chicago Press.

[CR49] Merli, C. E. M., Cetera, G. E., Caia, C., Facchin, F., & Vercellini, P. (2024). “The sound of silence” giving voice to endometriosis-related positional dyspareunia. *Archives of Gynecology and Obstetrics,**309*(3), 887–893. 10.1007/s00404-023-07205-3 37689593

[CR50] Mick, I., Freger, S. M., van Keizerswaard, J., Gholiof, M., & Leonardi, M. (2024). Comprehensive endometriosis care: A modern multimodal approach for the treatment of pelvic pain and endometriosis. *Therapeutic Advances in Reproductive Health,**18*, Article 26334941241277759. 10.1177/26334941241277759 39376635 PMC11457249

[CR51] Muise, A., Bergeron, S., Impett, E. A., & Rosen, N. O. (2017). The costs and benefits of sexual communal motivation for couples coping with vulvodynia. *Health Psychology,**36*(8), 819–827. 10.1037/hea0000470 28165264

[CR52] Myrtveit‐Stensrud, L., Haugstad, G. K., Rème, S. E., Schaller, S. L., & Groven, K. S. (2023). “It’s all my fault”: A qualitative study of how heterosexual couples experience living with vulvodynia. *Acta Obstetricia Et Gynecologica Scandinavica,**102*(10), 1378–1389. 10.1111/aogs.14537 36879489 PMC10540927

[CR53] Myrtveit-Stensrud, L., Schaller, S. L., Haugstad, G. K., & Groven, K. S. (2025). Navigating conflicting ideals of masculinity: A qualitative study of the experiences of male partners of women with vulvodynia. *Journal of Sex Research*, *62*, 1455–1466. 10.1080/00224499.2024.2371946 38958664

[CR54] Pais, A. S., & Almeida-Santos, T. (2023). Recent insights explaining susceptibility to endometriosis—From genetics to environment. *Wires Mechanisms of Disease,**15*(6), Article e1624. 10.1002/wsbm.1624 37533299

[CR55] Pazmany, E., Bergeron, S., Verhaeghe, J., Van Oudenhove, L., & Enzlin, P. (2014). Sexual communication, dyadic adjustment, and psychosexual well‐being in premenopausal women with self‐reported dyspareunia and their partners: A controlled study. *Journal of Sexual Medicine,**11*(7), 1786–1797. 10.1111/jsm.12518 24690206

[CR56] Pazmany, E., Bergeron, S., Verhaeghe, J., Van Oudenhove, L., & Enzlin, P. (2015). Dyadic sexual communication in pre-menopausal women with self-reported dyspareunia and their partners: Associations with sexual function, sexual distress and dyadic adjustment. *Journal of Sexual Medicine,**12*(2), 516–528. 10.1111/jsm.12787 25475508

[CR57] Perkins, A. (2019). The “silent” pain of endometriosis. *Nursing Made Incredibly Easy,**17*(3), 26–33. 10.1097/01.NME.0000554597.81822.03

[CR58] Pluchino, N., Wenger, J. M., Petignat, P., Tal, R., Bolmont, M., Taylor, H. S., & Bianchi-Demicheli, F. (2016). Sexual function in endometriosis patients and their partners: Effect of the disease and consequences of treatment. *Human Reproduction Update,**22*(6), 762–774. 10.1093/humupd/dmw031 27591248

[CR59] Queiroz, J. F., Aquino, A. C., Sarmento, A. C., Siqueira, B. B., Medeiros, H. D., Falsetta, M. L., Aquino, A. C. Q., Sarmento, A. C. A., Maurer, T., & Gonçalves, A. K. (2024). Psychosocial factors associated with vulvodynia. *Journal of Lower Genital Tract Disease*. 10.1097/LGT.0000000000000822 38697126

[CR60] Rancourt, K. M., Flynn, M., Bergeron, S., & Rosen, N. O. (2017). It takes two: Sexual communication patterns and the sexual and relational adjustment of couples coping with provoked vestibulodynia. *Journal of Sexual Medicine,**14*(3), 434–443. 10.1016/j.jsxm.2017.01.009 28189562

[CR61] Rancourt, K. M., Rosen, N. O., Bergeron, S., & Nealis, L. J. (2016). Talking about sex when sex is painful: Dyadic sexual communication is associated with women’s pain, and couples’ sexual and psychological outcomes in provoked vestibulodynia. *Archives of Sexual Behavior,**45*(8), 1933–1944. 10.1007/s10508-015-0670-6 26739823

[CR62] Romaioli, D., & Caprini, G. (2025). Narrating vulvodynia: A qualitative inquiry into experiences of chronic pain and the relevance of social relationships. *Qualitative Health Research*. 10.1177/10497323251357203 40754447

[CR63] Romaioli, D., Chiara, G., Faccio, E., & Miglietta, R. (2023). Hearing voices as a form of inner dialogue. Using the dialogical self to turn a critical voice into an ally. *Counselling Psychology Quarterly,**36*(3), 367–394. 10.1080/09515070.2022.2105306

[CR64] Romaioli, D., & McNamee, S. (2021). (Mis) constructing social construction: Answering the critiques. *Theory & Psychology,**31*(3), 315–334. 10.1177/09593543209677

[CR65] Rosen, N. O., Bergeron, S., Glowacka, M., Delisle, I., & Baxter, M. L. (2012). Harmful or helpful: Perceived solicitous and facilitative partner responses are differentially associated with pain and sexual satisfaction in women with provoked vestibulodynia. *Journal of Sexual Medicine,**9*(9), 2351–2360. 10.1111/j.1743-6109.2012.02851.x 22812596

[CR66] Rosen, N. O., Bergeron, S., Lambert, B., & Steben, M. (2013). Provoked vestibulodynia: Mediators of the associations between partner responses, pain, and sexual satisfaction. *Archives of Sexual Behavior,**42*, 129–141. 10.1007/s10508-012-9905-y 22350124

[CR67] Rosen, N. O., Bergeron, S., Sadikaj, G., Glowacka, M., Delisle, I., & Baxter, M. L. (2014). Impact of male partner responses on sexual function in women with vulvodynia and their partners: A dyadic daily experience study. *Health Psychology,**33*(8), 823. 10.1037/a0034550 24245835

[CR68] Rosen, N. O., Muise, A., Bergeron, S., Impett, E. A., & Boudreau, G. K. (2015). Approach and avoidance sexual goals in couples with provoked vestibulodynia: Associations with sexual, relational, and psychological well‐being. *Journal of Sexual Medicine,**12*(8), 1781–1790. 10.1111/jsm.12948 26176989

[CR69] Salvini A. (2011). *L’interazione semiotica in psicologia clinica*. In A. Salvini & M. Dondoni (Eds.), *Psicologia clinica dell’interazione e psicoterapia*, Giunti, Firenze

[CR70] Salvini, A. (2004). *Argomenti di psicologia clinica*. Padova: UPSEL Domeneghini.

[CR71] Salvini, A., & Dondoni, M. (2011). *Psicologia Clinica Dell’interazione E Psicoterapia* (pp. 416). Firenze: Giunti.

[CR72] Sametband, I., Chiara, G., Gaete-Silva, J., & Strong, T. (2024). Negotiating cultural relevance from within therapy conversations. *Human Systems,**4*(1), 3–20. 10.1177/26344041231199908

[CR73] Santangelo, G., Ruggiero, G., Murina, F., Di Donato, V., Perniola, G., Palaia, I., Fischetti, M., Casorelli, A., Giannini, A., Di Dio, C., Muzii, L., Benedetti Panici, P., & Bogani, G. (2023). Vulvodynia: A practical guide in treatment strategies. *International Journal of Gynecology & Obstetrics,**163*(2), 510–520. 10.1002/ijgo.14815 37154479

[CR74] Schick, M., Germeyer, A., Böttcher, B., Hecht, S., Geiser, M., Rösner, S., Eckstein, M., Vomstein, K., Toth, B., Strowitzki, T., Wischmann, T., & Ditzen, B. (2022). Partners matter: The psychosocial well-being of couples when dealing with endometriosis. *Health and Quality of Life Outcomes,**20*(1). 10.1186/s12955-022-01991-1 PMC9148469 35643578

[CR75] Sheppard, C., Hallam-Jones, R., & Wylie, K. (2008). Why have you both come? Emotional, relationship, sexual and social issues raised by heterosexual couples seeking sexual therapy (in women referred to a sexual difficulties clinic with a history of vulval pain). *Sexual and Relationship Therapy,**23*(3), 217–226. 10.1080/14681990802227974

[CR76] Simon, W., & Gagnon, J. H. (1986). Sexual scripts: Permanence and change. *Archives of Sexual Behavior,**15*(2), 97–120. 10.1007/bf01542219 3718206

[CR77] Simon, W., & Gagnon, J. H. (2003). Sexual scripts: Origins, influences and changes. *Qualitative Sociology,**26*(4), 491–497. 10.1023/B:QUAS.0000005053.99846.e5

[CR78] Smith, K. B., & Pukall, C. F. (2011). A systematic review of relationship adjustment and sexual satisfaction among women with provoked vestibulodynia. *Journal of Sex Research,**48*(2–3), 166–191. 10.1080/00224499.2011.555016 21409713

[CR79] Smith, K. B., & Pukall, C. F. (2014). Sexual function, relationship adjustment, and the relational impact of pain in male partners of women with provoked vulvar pain. *Journal of Sexual Medicine,**11*(5), 1283–1293. 10.1111/jsm.12484 24612656

[CR80] Stragapede, E., Huber, J. D., & Corsini-Munt, S. (2024). My catastrophizing and your catastrophizing: Dyadic associations of pain catastrophizing and the physical, psychological, and relational well-being of persons with endometriosis and their partners. *Clinical Journal of Pain,**40*(4), 221–229. 10.1097/AJP.0000000000001193 38229502

[CR81] Tayyeb, M., & Gupta, V. (2023). Dyspareunia. *StatPearls* [Internet]. https://www.ncbi.nlm.nih.gov/sites/books/NBK562159//

[CR82] Tomm, K. (1988). Interventive interviewing: Part III. Intending to ask lineal, circular, strategic, or reflexive questions? *Family Process,**27*(1), 1–15. 10.1111/j.1545-5300.1988.00001.x 3360095

[CR83] Tomm, K. (1989). *Systemic interviewing: a development of the therapeutic conversation*. Mareld.

[CR84] Tomm, K., George, S. S., Wulff, D., & Strong, T. (Eds.). (2014). *Patterns in interpersonal interactions: Inviting relational understandings for therapeutic change*. Routledge.

[CR85] Vowels, L. M., Roos, C. A., Mehulić, J., O’Dean, S. M., & Sánchez-Hernández, M. D. (2022). What does it mean to be responsive to a partner’s sexual needs? Toward a definition of sexual need responsiveness. *Archives of Sexual Behavior,**51*(8), 3735–3747. 10.1007/s10508-022-02432-2 36224303 PMC9663368

[CR86] Wahl, K. J., Imtiaz, S., Lisonek, M., Joseph, K. S., Smith, K. B., Yong, P. J., & Cox, S. M. (2021). Dyspareunia in their own words: A qualitative description of endometriosis-associated sexual pain. *Sexual Medicine,**9*(1), 100274–100274. 10.1016/j.esxm.2020.10.002 33291043 PMC7930843

[CR87] World Health Organization. (2023). https://www.who.int/news-room/fact-sheets/detail/endometriosis.

[CR88] Yong, P. J. (2017). Deep dyspareunia in endometriosis: A proposed framework based on pain mechanisms and genito-pelvic pain penetration disorder. *Sexual Medicine Reviews,**5*(4), 495–507. 10.1016/j.sxmr.2017.06.005 28778699

